# Paroxysmal dyskinesia and epilepsy in pseudohypoparathyroidism

**DOI:** 10.1002/mgg3.1423

**Published:** 2020-07-26

**Authors:** Chao Zhang, Xiangqin Zhou, Mei Feng, Wei Yue

**Affiliations:** ^1^ Department of Neurology Tianjin Medical University Tianjin China; ^2^ Department of Neurology Tianjin Huanhu Hospital Tianjin China; ^3^ Department of Neurology Peking Union Medical College Hospital Beijing China; ^4^ Department of Electrophysiology Tianjin Huanhu Hospital Tianjin China

**Keywords:** case report, epilepsy, paroxysmal kinesigenic dyskinesia, pseudohypoparathyroidism

## Abstract

**Background:**

Paroxysmal kinesigenic dyskinesia (PKD) and epilepsy share common pathogenic mechanisms but their pathophysiological connections remain unknown. Our study reports an individual with both disorders as a consequence of pseudohypoparathyroidism (PHP). This observation suggests potential shared pathophysiological mechanisms between PKD and epilepsy.

**Methods:**

We report the case of a 15‐year‐old male with pre‐diagnosed PKD and symptomatic epilepsy. We recorded the symptoms and carried out comprehensive biochemical, genetic, imaging, and EEG analyses to examine the characteristics and potentially shared etiology of these conditions.

**Results:**

In this case, the patient's PKD and symptomatic epilepsy were secondary to pseudohypoparathyroidism (PHP). The patient had a seven‐year history of intermittent, involuntary paroxysmal episodic movements, and a six‐year history of a loss of consciousness with convulsions. The electroencephalography results showed that the paroxysmal low and medium amplitude slow waves, isolated sharp waves, and sharp slow‐wave release occurred in the right prefrontal temporal cortex. Serum analysis indicated a calcium concentration of 1.91 mmol/L, a phosphorus concentration of 2.68 mmol/L, an alkaline phosphatase concentration of 114 IU/L, and a parathyroid hormone concentration of 109 pg/ml. Computerized tomography and magnetic resonance imaging results showed multiple calcifications in the bilateral frontal and parietal lobe cortex, bilateral thalamus, basal ganglia, and centrum semiovale. Furthermore, *GNAS* methylation abnormalities were discovered during methylation testing. There was no recurrence of abnormal movements or epileptic seizures, and calcium concentrations returned to healthy levels, following the pharmacological treatment of PHP.

**Conclusion:**

In this case, PKD and symptomatic epilepsy were caused by PHP. This report underscores the importance of looking for biochemical abnormalities in PKD and symptomatic epilepsy patients. We suggest that all such intractable epilepsy seizure patients should be screened for PHP.

## Graphical abstract

We summarized the pathophysiology and connection of paroxysmal kinesigenic dyskinesia (PKD) and epilepsy (EPI), and reported a patient with PKD and EPI was caused by pseudohypoparathyroidism, which is the first one that it had two seizure types: focal impaired awareness seizures and focal to bilateral tonic‐clonic seizures.

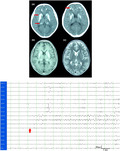

## 1 INTRODUCTION

The presentation of epilepsy in patients with paroxysmal kinesigenic dyskinesia (PKD) is a common occurrence. A previous study by Spacey et al., found that 10 of 15 (66.7%) PKD patients produced transient epileptic discharges during their clinical course (Spacey et al., 2002). In addition, researchers have studied the pathogenic mechanisms of PKD and epilepsy at the genetic level. Whilst both disorders may be caused by a number of pathogenic genetic mutations, a mutation in the pericentromeric region of chromosome 16 has been identified as a potential common cause of both (Biervert et al., 1998; Ma et al., 2018; Spacey et al., 2002). Furthermore, administration of the traditional anti‐epileptic drug (e.g., carbamazepine, lamotrigine, valproic acid, etc.) indirectly demonstrates that PKD and epilepsy may share common pathogenic mechanisms. In our study, we report a patient performing involuntary movements and experiencing symptomatic epileptic seizures consistent with the diagnoses of PKD and symptomatic epilepsy, respectively. Both conditions were secondary to pseudohypoparathyroidism (PHP) and this observation suggests a potential pathophysiological connection between the two secondarily observed disorders.

## 2 CASE PRESENTATION

The case history of our 15‐year‐old male patient revealed a seven‐year history of intermittent paroxysmal episodic involuntary movement and a six‐year history of episodes involving the loss of consciousness with convulsions. The involuntary movement, primarily in the right hemibody, presented as dystonia involving: the internal pronation and rotation of the shoulder joint; torsion and flexion of the wrist, knee, metacarpophalangeal, and interphalangeal joints; and tonic lower limb extension. During the episodes, the patient was conscious; however, he stared into the distance and experienced facial spasms, trismus, and aphasia. These conscious episodes lasted approximately 10 seconds and occurred up to 10 times per day before spontaneously resolving. And they could be induced by stretching the arms or legs.

Six years prior to admission, the patient developed a different seizure type in addition to the involuntary movement episodes. Prior to the onset of these seizures, he experienced warning symptoms (also known as an ‘aura’) including brain swelling or dizziness. These started with the repetitive and rigid act of chewing and swallowing. This was accompanied by a throwing motion of the right arm and a confused mental state. The duration of each episode was approximately 2–3 minutes and they occurred once or twice a month. During some of these episodes, the patient was found to have generalized tonic‐clonic seizures resulting in a loss of consciousness. This sometimes (2–3 times per year) led to him falling forwards. Records also showed an eight‐year history of tetany.

**Figure 1:**
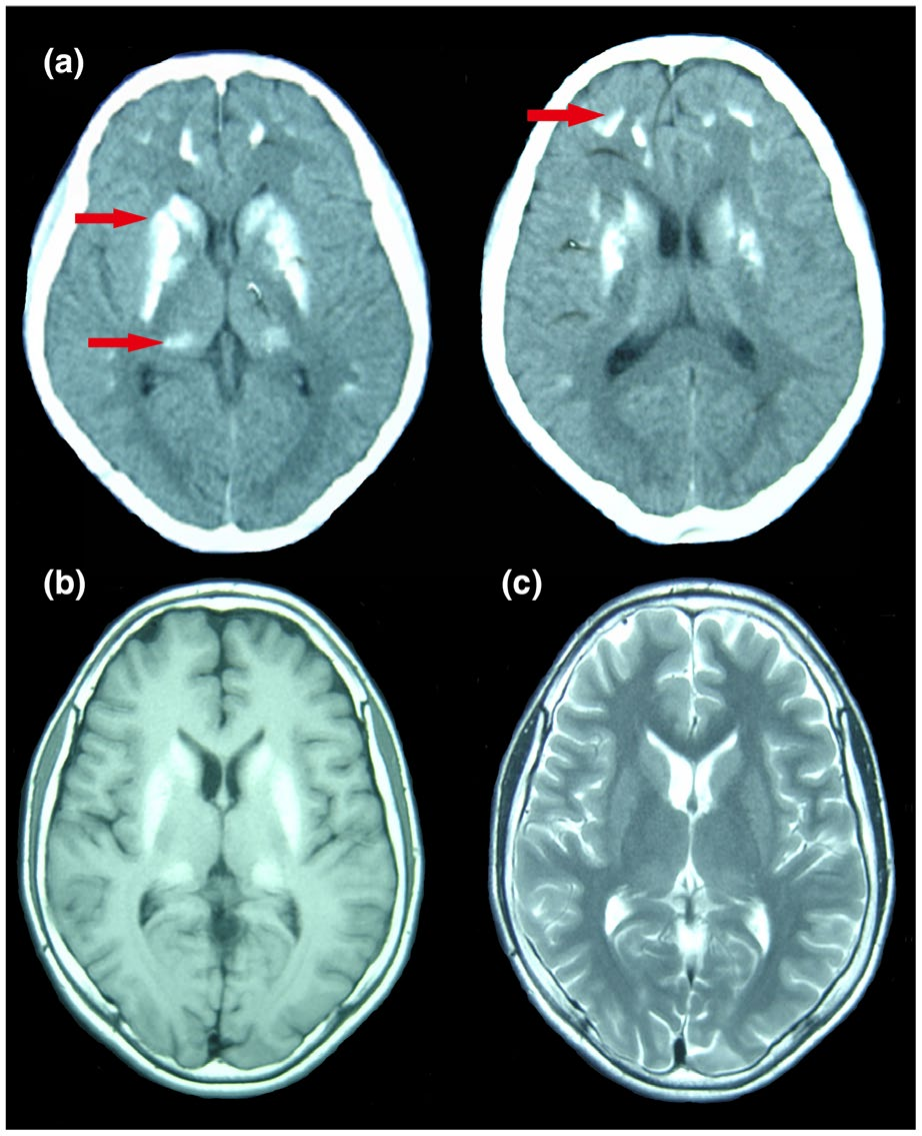
CT and MRI images of the patient's brain. (a) A CT image showing multiple calcifications bilaterally in the cortex of the frontal and parietal lobes, and the thalamus. Calcifications are also apparent in the basal ganglia and centrum semiovale. (b) Axial T1‐weighted MRI image showing hypointensities bilaterally in the cortex of the frontal and parietal lobes cortex, and the thalamus. Hypointensities are also apparent in the basal ganglia and centrum semiovale. (c) Axial T2‐weighted MRI image showing isointensities bilaterally in the cortex of the frontal and parietal lobes cortex, and the thalamus. Isointensities are also apparent in the basal ganglia and centrum semiovale

**Figure 2:**
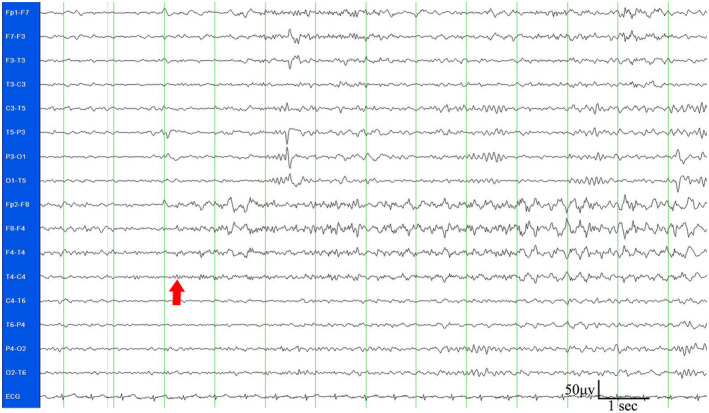
EEG results from an epileptic episode. The arrow indicates the point of seizure onset

The patient was 162 cm in height, weighed 54 kg. Apart from febrile convulsions at 6 months old, there was no history of neurological disease, brain tumor, or trauma. In his family history, there were no similar instances of involuntary movements. A physical examination was completed but revealed no typical positive signs. Serum analysis indicated a calcium concentration of 1.91 mmol/L (reference: 2.13–2.7 mmol/L), a phosphorus concentration of 2.68 mmol/L (reference: 0.74–2.7 mmol/L), an alkaline phosphatase concentration of 114 IU/L (reference: 42–390 IU/L), a parathyroid hormone (PTH) concentration of 109 pg/ml (reference: 7–13 pg/ml), and a urine calcium concentration of 1.6 mmol/24 h (reference: 2.7–7.5 mmol/24 h). Results for electrolyte concentration and markers of liver, kidney, and thyroid function were all within the acceptable ranges. Computerized tomography (CT) and magnetic resonance imaging (MRI) imaging of the brain revealed bilateral calcium deposition in the cortex of the frontal and parietal lobes, and in the thalamus (Figure 1). Electroencephalography (EEG) data showed paroxysmal low and medium amplitude slow waves, isolated sharp waves, and sharp slow wave release in the right prefrontal temporal lobe (Figure 2, supporting Figure S1). Neither mutations nor copy number variations were detected in the genes of interest during genetic analysis (eg.*GNAS*, *STX16*, *GNAS*‐*AS1*, etc. [Reference Sequence: NC_000020.11]); however, *GNAS* methylation abnormalities were present. No abnormalities were found during an abdominal B‐mode ultrasound or following an X‐ray of the hands.

Following treatment with carbamazepine (400 mg/day), calcium supplements (1000 mg/day) and vitamin D (10,000 U/day), there was no recurrence of abnormal movements or seizures during a 3‐month follow‐up period and the blood calcium returned to normal.

## 3 DISCUSSION

Paroxysmal dyskinesia (PD) is a group of intermittent but recurrent neurological movement disorders with rare motor manifestations. PD can be classified into four sub‐groups according to its precipitating factors (Erro, 2016). PKD is the most common form and manifests as brief episodes of abnormal movement that are triggered by sudden voluntary movements, most episodes last 10–30 seconds and can involve both the hemi and whole body without a loss of consciousness (Yin et al., 2018; Zhao et al., 2020).

Brain calcifications have been reported in 42.6–100% of patients with PHP; however, these may be incidental findings, particularly in older individuals, that these brain calcifications may not be because of neurological diseases (Deng, Zheng, & Jankovic, 2015). Cerebral perfusion single‐photon emission computed tomography (SPECT) studies have revealed brain calcifications in the bilateral caudate nuclei which supports the theory that abnormal basal ganglia (BG)–thalamocortical circuits are present in PKD patients (Joo et al., 2005). Furthermore, numerous studies have demonstrated that the BG plays an important part in epilepsy propagation and modulation. Whilst some have argued that the BG is a seizure propagation pathway that can suppress EEG signatures and behavioral expression of epileptic seizures, others argue that the BG circuitry can remotely inhibit epileptic seizures (Kim et al., 2016). Carrying these theories forward, calcifications in the BG may disrupt inhibitory circuits thus causing patients to experience epileptic seizures. Up until now, we were not able to demonstrate that these cortical deposits are directly related to epileptogenicity.

Our patient was diagnosed with PKD and symptomatic epilepsy both of which were secondary to, and caused by, PHP. His symptomatic epilepsy consisted of two seizure types: focal impaired awareness seizures and focal to bilateral tonic‐clonic seizures in accordance with the International League Against Epilepsy (ILAE) 2017 classifications (Scheffer et al., 2017). PHP is rare and heredity hormone resistance syndrome first reported in 1942 by Albright et al. The pathophysiology of PHP involves the dysfunction of parathyroid hormone (PTH) receptors in peripheral target organs or in their signal transduction pathways (Mantovani, 2011). There are two types of PHP which are classified according to the reaction of renal proximal convoluted tubules to bovine PTH extract and characterized by the PTH resistance via the deficiency of glutamine synthase (GS) activity in various cell types, which is different with hypoparathyroidism in the high or normal serum PTH level. And up to now, lots of the mutation in the gene encoding the Gsα (GNAS) was reported (Mantovani et al., 2018). The patient presented with abnormal activities after the strenuous exercise. These extremely intensive kinesigenic stimuli caused conduction through the subcortical thalamic nuclei and basal ganglia to neurons resulting in seizure episodes (Yin et al., 2018). Gamma‐aminobutyric acid (GABA), a critical inhibitory neurotransmitter that reduces neuronal excitation in the brain, is influenced by the extracellular concentration of calcium ions. A reduction in this concentration decreases the probability of GABA‐containing synaptic vesicles being released which, in turn, disinhibits the postsynaptic potential. This is in addition, and secondary to, the overactivation of dopamine in the striatum (Wang et al., 2017). We propose that the patient's hypocalcemia in combination with intensive kinesigenic stimuli, stimulated the thalamus thus causing repeated bursts of thalamic‐cortical action potentials and the subcortical–cortical spread. To simplify, PHP, which is associated with hypocalcemia, hyperphosphatemia, and raised PTH concentrations, leads to the dysfunction of G proteins which can alter renal calcium resorption thus decreasing GABAergic inhibition. This increased excitability results in PKD or epilepsy seizures (Ritter et al., 2015).

PHP is a rare cause of PKD. Previous to our study, only two articles mentioned PKD and epilepsy caused by PHP (Huang, Chen, & Tsai, 2005; Prashantha & Pal, 2009). In this report, the patient had diagnoses of both PKD and symptomatic epilepsy which was composed of two seizure types. Both of these conditions were secondary to the diagnosis of PHP and this observation underscores the importance of looking for biochemical abnormalities. This suggests that all such patients with intractable epileptic seizures should be screened for PHP.

## 4 CONCLUSION

Our study describes the case of a 15‐year‐old male with PKD and symptomatic epilepsy caused by PHP. It reports his clinical symptoms, genetic test results, and the EEG, CT, and MRI findings. This highlights the importance of looking for biochemical abnormalities in patients with intractable epilepsy. We suggest that all such patients should be screened for PHP.

## CONFLICT OF INTEREST

The authors declare that they have no conflicts of interest. The patient gave his informed consent for us to include his details in this report.

## AUTHOR CONTRIBUTIONS

Chao Zhang data assembly and manuscript preparation; Xiangqin Zhou clinical analysis about the case; Mei Feng analyzed the EEG; Wei Yue came up with the core idea for the study and final approval of the paper.

## Supporting information

Fig S1

Video S1
