# Supplementary figures and images for: Paroxysmal dyskinesia and epilepsy in pseudohypoparathyroidism
Source: Mol Genet Genomic Med. 2020 Jul 26;8(10):e1423. doi: 10.1002/mgg3.1423 (PMC7549567; doi:10.1002/mgg3.1423)

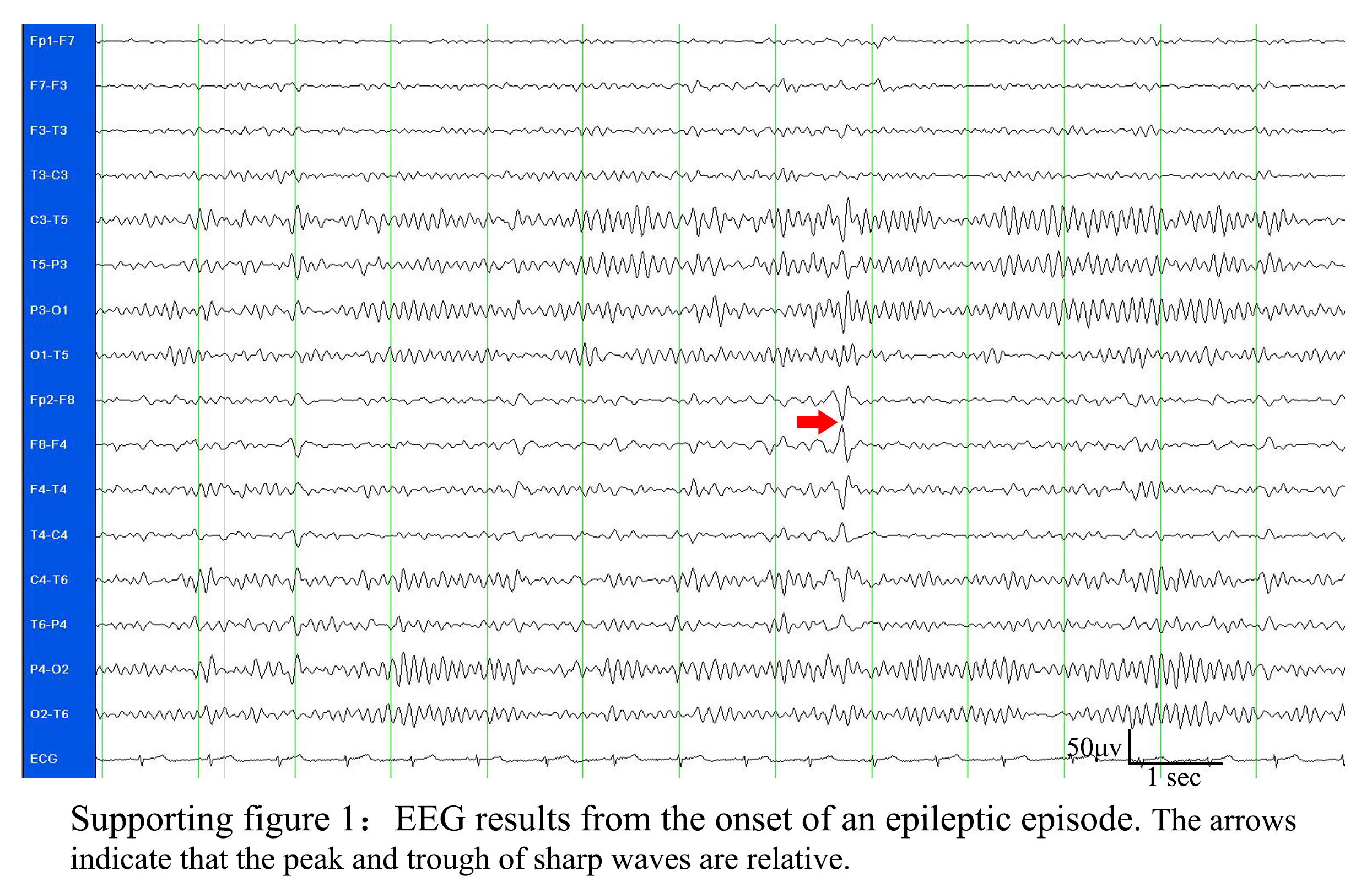

Supplement: Supplementary file 1 — Fig S1 [file MGG3-8-e1423-s001.tif]
